# Long‐Acting Ocular Injectables: Are We Looking In The Right Direction?

**DOI:** 10.1002/advs.202306463

**Published:** 2023-11-28

**Authors:** Mickael Dang, Molly S. Shoichet

**Affiliations:** ^1^ Department of Chemical Engineering and Applied Chemistry University of Toronto 200 College Street Toronto ON M5S 3E5 Canada; ^2^ Donnelly Centre for Cellular and Biomolecular Research University of Toronto 160 College Street Toronto ON M5S 3E1 Canada; ^3^ Institute of Biomedical Engineering 164 College Street Toronto ON M5S 3G9 Canada

**Keywords:** hydrogels, injectable implants, long‐acting injectables, ocular chronic diseases, sustained drug release kinetics, targeted nanocarriers

## Abstract

The complex anatomy and physiological barriers of the eye make delivering ocular therapeutics challenging. Generally, effective drug delivery to the eye is hindered by rapid clearance and limited drug bioavailability. Biomaterial‐based approaches have emerged to enhance drug delivery to ocular tissues and overcome existing limitations. In this review, some of the most promising long‐acting injectables (LAIs) in ocular drug delivery are explored, focusing on novel design strategies to improve therapeutic outcomes. LAIs are designed to enable sustained therapeutic effects, thereby extending local drug residence time and facilitating controlled and targeted drug delivery. Moreover, LAIs can be engineered to enhance drug targeting and penetration across ocular physiological barriers.

## Graphical abstract

This review focuses on innovative strategies to deliver therapeutics directly to the eye. By designing novel injectable materials with slow release properties, therapeutics are released directly to the eye for a prolonged period of time, thereby providing greater efficacy with reduced side effects.

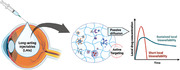

## 1 Introduction

### 1.1 *Long‐Acting* is Defined by Extended Therapeutic Efficacy

Long‐acting injectables (LAIs) for ocular drug delivery provide sustained drug release over an extended period; however, the duration of action differs based on the targeted ocular tissue, injection site, and therapeutic objectives.^[^ ^1^, ^2^, ^3^ ^]^ For example, an LAI targeting the anterior segment, such as the cornea or aqueous humor, may exhibit a different duration of action compared to a similar injectable targeting the posterior segment, like the vitreous humor or retina, due to their distinct clearance rates and physicochemical properties.^[^ ^4^ ^]^ Amo et al. demonstrated that an intravitreal (posterior segment) injection of a small molecule drug, such as brimonidine, increases its vitreous residence time by one order of magnitude compared to an intracameral (anterior segment) injection.^[^ ^5^ ^]^ Thus, there is no uniform definition for *long‐acting* across the entire eye.

**Figure 1:**
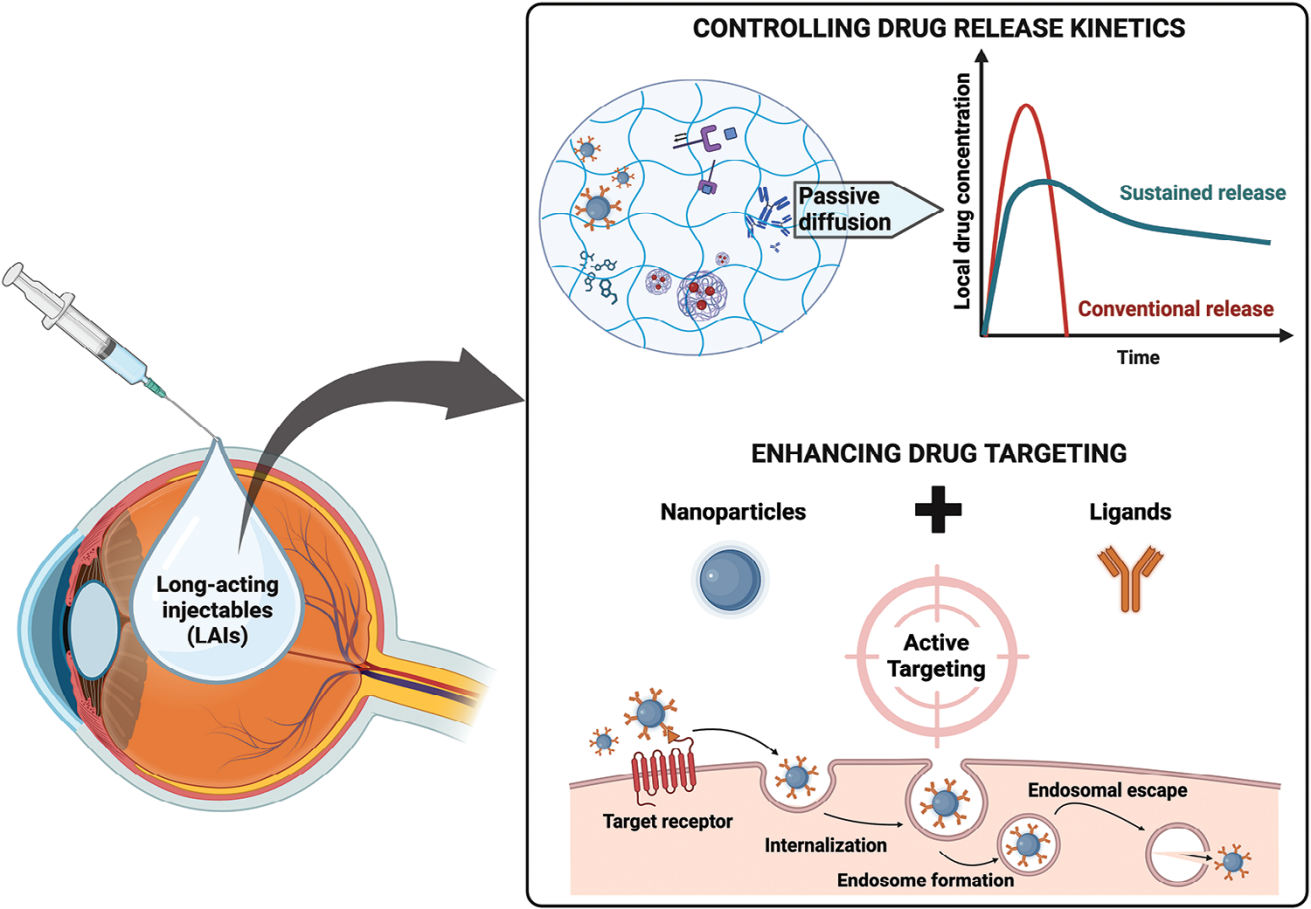
Ocular LAIs – Passive diffusion versus active targeting. A comparison between passive diffusion‐based drug release kinetics, where therapeutics disperse and release from the delivery system gradually (top) and active targeting strategies with drug molecules specifically directed toward their intended target for increased therapeutic efficacy (bottom).

*Long‐acting* can also include targeted delivery with the goal of precisely delivering therapeutics to specific tissues or cells within the eye.^[^ ^6^, ^7^ ^]^ Thus, *long‐acting* includes both prolonged drug release and maintaining therapeutic drug levels at the target site for an extended period, thereby enhancing treatment efficacy (**Figure** **1**).^[^ ^7^ ^]^

The *long‐acting* behavior of an LAI depends on the vehicle used for delivery. Some LAI vehicles are biostable whereas others are bioresorbable: they efficiently transport therapeutic agents to the intended ocular cells, enabling sustained drug exposure and therapeutic effect.^[^ ^8^ ^]^ Standardizing the criteria for what constitutes long‐acting based on the intended site of injection and specific therapeutic objectives is an important consideration in ocular drug delivery. For example, in the case of topical ophthalmic solutions, an extension of drug action from several h to days is regarded as long‐acting given the rapid tear fluid clearance and regular blinking. Liu et al. formulated mucoadhesive dextran‐based nanoparticles using phenylboronic acid to treat anterior eye diseases and demonstrated long‐acting behavior with prolonged drug bioavailability up to 5 days.^[^ ^9^ ^]^ Conversely, in the case of a drug depot placed within an ocular cavity, it is often considered long‐acting only when it remains effective for at least one week or even one month, primarily due to the invasiveness of the surgical procedure involved. Durysta, an intracameral implant, has been designed to prolong the release of a small‐molecule drug for at least 15 weeks due to the potential invasiveness of the injection.^[^ ^10^ ^]^ As the field advances and novel delivery technologies emerge, a critical reassessment of the implications of this term is necessary to ensure an accurate representation and understanding of the actual therapeutic benefits these formulations offer to patients with ocular diseases.

The development of LAIs has transformed ocular drug delivery, offering significant advantages over conventional strategies. These novel formulations aim to prolong therapeutic efficacy, reduce dosing frequency, and ultimately improve patient compliance for multiple chronic ocular pathologies by providing sustained drug release.^[^ ^11^ ^]^ A key advantage of LAIs is their ability to maintain consistent therapeutic levels at the target site, overcoming the limitations of conventional short‐acting formulations.^[^ ^1^ ^]^ Specifically, these injectables minimize peaks and troughs in drug concentration, thereby minimizing off‐target side effects such as systemic toxicity.^[^ ^11^ ^]^ This prolonged drug exposure allows for less frequent administration and enhances patient convenience and compliance, particularly in the management of chronic ocular conditions, such as glaucoma that requires patients to self‐administer daily ophthalmic drops that can cause blurred vision and systemic toxicity. Several Food and Drug Administration (FDA)‐approved LAI formulations have already shown promise in addressing conditions like glaucoma, age‐related macular degeneration, and several inflammatory ocular disorders.^[^ ^12^ ^]^ LAIs have been widely investigated for both small molecule drugs and biologics to the eye. Biologic agents, including antibodies and growth factors, often require frequent injections to maintain therapeutic effects due to their inherent instability.^[^ ^13^ ^]^ Thus, LAIs present an opportunity to reduce the injection burden while ensuring protein stability and sustained bioavailability.

This review focuses on the engineering considerations essential for the development of some of the most promising LAIs in ocular drug delivery and complements previous reviews, which have discussed drug delivery technologies that address limitations in current clinical ocular treatments.^[^ ^14^, ^15^ ^]^

### 1.2 Intraocular Versus Periocular Injections

**Figure 2:**
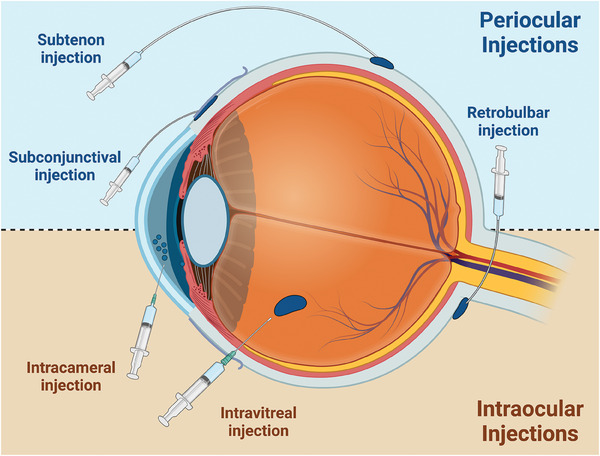
Overview of commonly used ocular injections for LAIs. The various injection routes frequently employed for administering LAIs. These routes include intravitreal, intracameral, subtenon, subconjunctival, and retrobulbar injections, each offering distinct pharmacokinetic profiles and absorption rates for sustained drug release over extended periods.

Researchers have investigated the intricate interplay between administration routes and the therapeutic effectiveness of LAIs. Specifically, the route of administration plays a central role in both shaping key pharmacokinetic parameters – including drug absorption, distribution, and overall bioavailability – and the engineering design for an ideal LAI. Generally, researchers have extensively explored two routes for local administration: intraocular and periocular injection (**Figure** **2**). Each approach has distinct merits and drawbacks, which significantly influence their utility for different therapeutics and diseases.

Intraocular injection involves the direct administration of therapeutics into the interior of the eye. This technique circumvents external ocular barriers, such as the outer blood‐retina barrier, and enables delivery to either the vitreous humor (the clear gel‐like fluid within the eye) or the front chamber of the eye.^[^ ^16^ ^]^ Intravitreal injections have become common to manage retinal diseases such as neovascular age‐related macular degeneration (nAMD), also referred to as wet AMD, diabetic retinopathy, and retinal vein occlusions.^[^ ^17^ ^]^ For example, intravitreal administration of anti‐vascular endothelial growth factor (anti‐VEGF) antibodies or corticosteroids, is typically used to mitigate abnormal blood vessel growth and inflammation in the retina.^[^ ^18^ ^]^ In some cases, therapeutics are introduced intracamerally, into the fluid‐filled space between the cornea and the iris, though this method is less prevalent and primarily used to target the trabecular meshwork cells in glaucoma.^[^ ^19^, ^20^ ^]^ Intraocular injections allow the delivery of therapeutics directly to the site of action, minimizing systemic side effects typically linked with periocular treatments. These injections necessitate invasive procedures and skilled administration to mitigate potential risks such as acute inflammation and other surgical‐induced symptoms (retinal detachment, hypertension, etc.).^[^ ^21^, ^22^, ^23^ ^]^

Periocular injections target the tissues surrounding the eye. This strategy permits localized treatment of ocular conditions while obviating the need for invasive, intraocular injections.^[^ ^24^ ^]^ In subconjunctival injection, for example, drugs are delivered below the conjunctiva – a thin membrane covering the white portion of the eye and inner eyelids. This procedure is often used to treat corneal inflammation, infections, and specific eye allergies.^[^ ^25^ ^]^ With subconjunctival delivery, drugs target the ocular surface and potentially penetrate deeper tissues, including the scleral stroma and the ciliary body.^[^ ^26^ ^]^ Similarly, sub‐tenon injections –, i.e., into the tenon capsule – are commonly used to manage conditions in the posterior eye segment, such as uveitis or macular edema, thereby achieving local effects.^[^ ^27^ ^]^ While less frequent, retrobulbar injections are employed for conditions like optic neuritis, targeting the optic nerve and its surroundings.^[^ ^28^ ^]^ Periocular injections are less invasive than intraocular injections; however, they do not allow treatment of retinal diseases due to the limitations in diffusion across ocular tissues.

The choice of route of administration, whether intraocular or periocular, impacts the effectiveness and safety of the treatment, as summarized in **Table** **1**.

**Table 1:** Route of administration effects outcome for LAIs.

Consideration	Intraocular	Periocular	Reference
Diffusion path and concentration	A more direct path for the drug to reach target tissues within the eye, potentially resulting in higher concentrations therein	Drug diffuses across the various cell layers before reaching the target tissue, which may lead to lower drug concentrations and a slower onset of action	[29]
Space and volume	Typically requires a smaller volume of injectable due to the limited capacity of the vitreous cavity, necessitates a non‐swelling injectable biomaterial.	Larger volumes can be administered due to greater available space surrounding the eye; however, this can lead to greater dilution and potentially less localization of the drug.	[30]
Frequency and invasiveness	Standard of care for retinal diseases, such as wet AMD, due to their outstanding clinical benefit despite their invasiveness and (bi)‐monthly administration.	Standard of care for diseases such as glaucoma, despite multiple daily administration, due to the convenience of ophthalmic eye drops.	[11]

Ultimately, the decision between intraocular and periocular administration depends on factors including the pharmacokinetics of the drug, the specific anatomy of the eye, the target tissue, the desired treatment duration, and the acceptable level of risk.

### 1.3 Challenges of Traditional Ocular LAIs

Generally, sustained delivery of ocular therapeutics has shown promise in improving treatment efficacy, minimizing side effects, and reducing challenges related to patient compliance, especially in chronic diseases. Age‐related chronic conditions, such as glaucoma and AMD, are leading causes of irreversible blindness.^[^ ^31^ ^]^ Recent advances in intraocular injectables for sustained drug release demonstrate the growing importance of next‐generation approaches for ocular disease management and include: Durysta – an intracameral implant for continuous intraocular pressure (IOP) lowering for glaucoma;^[^ ^10^ ^]^ and Susvimo – an LAI for continuous intravitreal delivery of ranibizumab.^[^ ^32^ ^]^ Notwithstanding these advances, issues such as excipient material buildup, the need for device removal, and the potential for foreign body reactions can further complicate treatment outcomes, as summarized:^[^ ^33^ ^]^

- **Lack of flexibility in dosage adjustment**: LAIs provide sustained drug release over extended periods, which can limit the ability to adjust dose based on individual patient response or changing disease conditions. In some cases, patients may require personalized dosage regimens, and LAIs might not accommodate such adjustments effectively.^[^ ^34^ ^]^ For example, numerous corticosteroids increase IOP, thus the dose is monitored and adjusted by IOP‐lowering therapeutics.^[^ ^35^ ^]^ Moreover, if unexpected adverse effects or complications arise, it may be challenging to swiftly address or reverse the drug's action.^[^ ^36^ ^]^ Thus, having an easily retrievable LAI could serve as an asset, allowing healthcare professionals to adjust dosage over time to meet the growing demands of patients while maintaining the therapeutic benefits of extended drug release.
- **Increased risk of non‐compliance**: Despite the advantage of reduced dosing frequency, LAIs can inadvertently lead to treatment non‐compliance when balanced with ease of use. Patients might perceive the extended duration between injections as an opportunity to skip or delay follow‐up appointments, compromising treatment efficacy.^[^ ^11^ ^]^ Thus, designing LAIs based on the likely dosing habits a patient would adopt will be crucial to the clinical translation of these technologies.

To overcome these challenges and advance ocular disease management, novel approaches are required to enhance the efficacy, safety, and patient experience associated with ocular LAIs. Specifically, two promising avenues to optimize therapeutic efficacy are being pursued:

1. **Enhancing drug properties to bolster their pharmacological effects**. Specifically, researchers have engineered biologics and small‐molecule drugs to improve bioavailability, extend half‐life, and enhance tissue penetration. These tailored modifications can significantly elevate the therapeutic potential of these agents. For example, optimizing the structure of a specific monoclonal antibody could enhance its ability to bind to targeted receptors in the eye, thereby improving the efficacy in treating numerous ocular pathologies.^[^ ^37^ ^]^
2. **Leveraging injectable attributes**. Another avenue focuses on harnessing the physicochemical attributes of injectables to enhance local drug residence time and ensure consistent drug availability. Specifically, injectables enable sustained release, ensuring consistent therapeutic dosing at the site of action while minimizing systemic exposure and potential off‐target side effects. For example, an injectable formulation of a glaucoma medication may incorporate microspheres to gradually release the drug over several months, maintaining IOP within the desired range without the need for frequent administrations.^[^ ^38^ ^]^

These approaches present remarkable opportunities for advancing drug therapies and addressing critical medical challenges with superior efficacy and ultimately patient outcomes.

## 2 Enhancing Chemical Properties of Ocular Bioactive Molecules

By manipulating the chemical attributes of ocular therapeutics, researchers seek to achieve improved solubility, stability, and bioavailability, thereby augmenting their overall efficacy.

### 2.1 Drug Conjugates Overcome Ocular Cell Membrane Barriers

**Figure 3:**
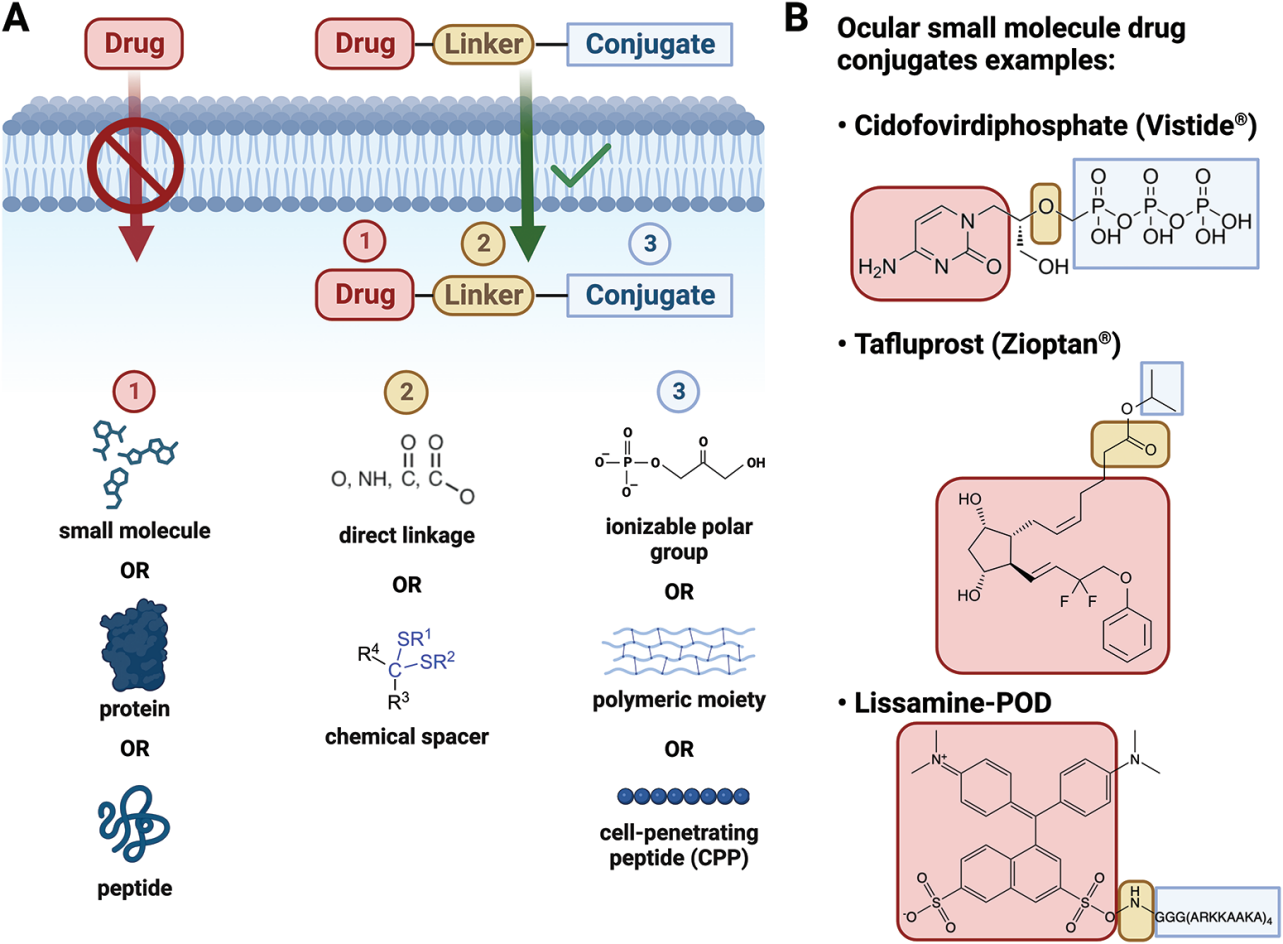
Drug conjugates can overcome ocular physiological barriers to reach their therapeutic targets. A) Drugs are typically linked to conjugates through either a direct linkage or a chemical spacer. Conjugates including ionizable polar groups, polymeric moieties and cell‐penetrating peptides can be utilized to enhance drug permeability throughout ocular cell membranes. B) Notable examples of ocular small molecule drug conjugates (i.e., cidofovirdiphosphate, tafluprost and lissamine‐POD).

Drug solubility is key for efficacy and has been achieved with pH adjustment and/or incorporation of surfactants or other excipients;^[^ ^39^, ^40^ ^]^ however, these strategies often result in tissue irritation or toxicity.^[^ ^41^ ^]^ Drug conjugation strategies have been explored for parenteral administration and typically include a polar functional group, such as ionizable moieties, to enhance water solubility (**Figure** **3**A). Ionizable polar moieties often result in significantly higher solubility than non‐ionizable ones, like polyethylene glycol (PEG).^[^ ^42^ ^]^ Consequently, many commercially available parenteral drug conjugates incorporate ionizable groups, such as succinic acid, phosphate, or ammonium groups. For example, cidofovir (CDV) diphosphate, (brand name Vistide) is an intracellularly active molecule conjugated with phosphate groups and used for the treatment of a broad‐range of DNA virus infections, including cytomegalovirus (CMV) retinitis in people with AIDS (Figure 3B).^[^ ^43^ ^]^ While injected intravitreally following retinal surgery, these soluble small‐molecule drugs cannot cross retinal cell membranes, which hinders their therapeutic effect. The limited ability of polar and charged drugs to cross biological membranes remains a significant challenge.^[^ ^38^ ^]^ Even following local application, drugs with poor permeability display limited levels of exposure within target cells. These properties have constrained the application of this approach in recent prodrug development.

Enhancing membrane permeability is key to prodrug research and has yielded substantial advancements to date.^[^ ^45^, ^46^ ^]^ A prodrug is a precursor molecule that is converted into an active form after administration. In ocular applications, various metabolic processes, such as enzymatic hydrolysis (e.g., with corneal esterases), are used to convert a prodrug to its active form, which can be tuned for specific control over the release at the target site.^[^ ^47^, ^48^ ^]^ Several prodrugs have been synthesized with lipid moieties to enhance cell membrane permeability after ocular administration to increase therapeutic efficacy.^[^ ^49^ ^]^ Lipid‐based prodrugs, including small‐molecules and protein biologics, often include a chemical spacer to further improve bioavailability or tune drug release kinetics, by, for example, the use of either a bio‐cleavable or stimulus‐responsive linker.^[^ ^50^ ^]^

Modulating the chemical properties of the drug, lipid or spacer impacts the in vivo fate of the active compound. For example, brincidofovir, a cidofovir prodrug bearing a hexadecyloxypropyl cleavable group (HDP‐CDV), has been investigated as a long‐lasting, intravitreal, antiviral ocular drug. Wang et al. demonstrated the safety of HPD‐CDV in New Zealand Red rabbit eyes for at least 30 weeks.^[^ ^44^ ^]^ Additionally, intravitreal pharmacokinetic studies of HDP‐CDV in rabbit eyes demonstrated an extended drug half‐life of 8.4 days in the retina compared to an unmodified CDV of 2.6 h.^[^ ^44^ ^]^ While already FDA‐approved in 2021 for the treatment of smallpox (as Tembexa) HPD‐CDV is currently being evaluated in a Phase III clinical trial for the treatment of CMV retinitis.^[^ ^51^ ^]^ Interestingly, tafluprost (brand name Zioptan) is a prodrug of tafluprost acid, which is an analog of prostaglandin F2α and acts as an agonist for human prostaglandin F receptor, managing elevated intraocular pressure in cases of glaucoma. Due to the lipophilic isopropyl ester, tafluprost efficiently traverses the cornea upon periocular administration.^[^ ^52^ ^]^ Ocular carboxylesterases predominantly hydrolyze tafluprost into tafluprost acid, thus ensuring its intraocular pressure‐lowering effects up to 24 h after application. Numerous other prodrugs, designed as analogs of prostaglandin F2α, exhibit potent and long‐lasting intraocular pressure reduction.^[^ ^52^ ^]^ These compounds, such as isopropyl esters like latanoprost and travoprost as well as the ethanolamide‐based prodrug bimatoprost, are widely employed as front‐line treatments for glaucoma and ocular hypertension. While numerous prodrugs have already found their way to the clinic, challenges of limited ocular bioavailability due to high hydrophobicity remain.^[^ ^53^ ^]^ Thus, striking the right balance between solubility and membrane permeability is a complex but crucial aspect of ocular drug design. It is often a trade‐off, where increasing a drug's solubility might decrease its lipophilicity and vice versa.

Cell‐penetrating peptide (CPP)‐drug conjugates have shown promise in delivering numerous ocular therapeutics, including biologics, small molecules, and peptides.^[^ ^54^ ^]^ Typically, CPPs consist of fewer than 30 amino acid residues, with a notable abundance of basic amino acids, such as arginine and lysine. They transport a wide range of cargo across ocular cellular membranes while retaining their functional integrity.^[^ ^55^ ^]^ CPPs were inspired by natural penetration domains found in viruses, thus allowing the rational design for novel synthetic ones.^[^ ^56^ ^]^ Johnson et al. designed the first CPP for ocular delivery, named POD (GGG(ARKKAAKA)_4_, ≈3.5 kDa), for posterior segment diseases, including AMD and retinitis pigmentosa. Specifically, POD was conjugated to a vital water‐soluble dye, lissamine green (often used for ocular examination), to form lissamine‐POD (L‐POD). Following intravitreal injection of L‐POD in mice, more than 85% of lissamine was transduced within the first 2 h and accumulated within the outer nuclear layer (ONL) and retinal pigment epithelium (RPE) for up to 20 h. In contrast, injection of lissamine alone demonstrated significantly lower or no accumulation in the retinal layers after 20 h compared to L‐POD.^[^ ^57^ ^]^ Thus, POD may be useful for the treatment of posterior segment diseases, such as age‐related macular degeneration or retinitis pigmentosa.

Alternatively, Nguyen et al. explored the use of CPPs in conjunction with polymeric nanoparticles. They created nanotherapeutics through aminolysis of resveratrol‐encapsulated polycaprolactone nanoparticles (R@PCL NPs), followed by the formation of amide linkages with carboxyl‐terminated transactivator of transcription CPP (T) and metformin (M). These R@PCL‐T/M NPs demonstrated prolonged drug release, ocular biocompatibility, and bioactivity to address retinal disease risk factors in vitro. In vivo studies in rats revealed that a single intravitreal dose of R@PCL‐T/M NPs significantly improved retinal permeability (by ≈15‐fold), preserved endogenous antioxidants, and inhibited abnormal vessel growth in the retina for at least 56 days.^[^ ^58^ ^]^

Additionally, CPPs have enabled long‐acting periocular delivery of therapeutics, which were originally designed for intraocular administration, via transcytosis,^[^ ^59^ ^]^ thus decreasing invasiveness of treatment and improving patient compliance. Cogan et al. conjugated oligoarginine CPPs (RRRRRR) to large negatively charged antibodies, including ranibizumab (Lucentis) and bevacizumab (Avastin) for the treatment of wet AMD. They demonstrated sustained therapeutic dose levels of ranibizumab and bevacizumab up to 24 h in porcine eyes, thus allowing a daily dosing regimen through topical administration, instead of monthly, intravitreal injections in the clinic.^[^ ^60^ ^]^ While daily administration would typically be more cumbersome for patients, the less invasive, self‐administered topical has advantages over the monthly intravitreal injection of therapeutics that require a surgeon. Although significant advancements have been made in technologies that enhance cell penetration, no CPP‐conjugated ocular therapeutics have been approved by the FDA to date, likely due to the lack of both understanding of their immunogenicity and specificity in vivo.^[^ ^61^ ^]^

### 2.2 Greater Ocular Cell Targeting, Fewer Off‐Target Side Effects

**Figure 4:**
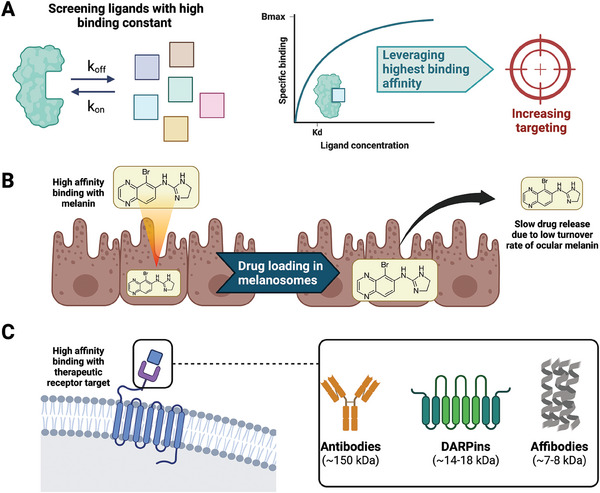
Leveraging high binding affinity can enhance ocular therapeutic targeting. A) Highest affinity for distinct proteins can be determined by screening different binding ligands to increase drug targeting. B) Certain ocular therapeutics, such as brimonidine, show high binding affinity for melanin ‐ a pigment abundant in retinal pigmented epithelial cells with a slow turnover rate. This high affinity enables these drugs to be sequestered by melanin‐containing cells, resulting in a localized and prolonged drug release at the intended target site. C) Various molecular constructs, including antibodies, DARPins, and affibodies, can be strategically designed to exploit their high affinity for specific target receptors, thus enhancing therapeutic localization.

To increase long‐acting therapeutic effects, drugs can be designed to target specific receptors in the eye. While certain active compounds exhibit high efficacy against ocular diseases in controlled environments, their clinical viability is often compromised by their brief retention in ocular tissues and the absence of targeted delivery mechanisms.^[^ ^62^ ^]^ Rapid turnover of ocular fluids limits the duration of action, and non‐specific distribution hinders therapeutic impact. Targeted carriers aim to enhance localized delivery, thereby unlocking the potential of these compounds for effective treatment in clinical settings (**Figure** **4**A).^[^ ^62^ ^]^

Binding to melanin, a pigment present within melanosomes in various ocular tissues such as the retinal pigment epithelium (RPE) and the uveal coat, enhances the distribution of ocular drugs.^[^ ^63^ ^]^ Given the low turnover rate of ocular melanin, a drug that binds to melanin might accumulate in pigmented eye tissues, potentially leading to either drug toxicity or sequestration.^[^ ^64^ ^]^ With a suitable balance of binding affinity and capacity, melanin could serve as a depot for sustained drug release within the eye, ultimately resulting in an extended period of therapeutic effectiveness (Figure 4B). Several drugs have exhibited inherent binding to melanin via either physical adsorption (e.g., hydrogen bonding, van der Waals forces, etc.) or chemical bonding, which can extend the pharmacological activity within the eye.^[^ ^65^ ^]^ Hsueh et al. developed a machine‐learning methodology to engineer next‐generation targeting peptides that efficiently enter ocular cells and bind to melanin while preventing cytotoxicity. Specifically, they demonstrated that brimonidine, an alpha‐agonist used for the treatment of chronic glaucoma, conjugated with an engineered peptide HR97 (FSGKRRKRKPR) improved, by ≈17 fold, the cumulative IOP‐lowering effect compared to free injection over a period of 20 days.^[^ ^66^ ^]^ Although melanin binding is promising for maintaining therapeutic levels for a prolonged period, the inherent variability in melanin levels in individuals poses a challenge.^[^ ^64^, ^67^ ^]^ Melanin content in ocular cells can differ significantly among individuals due to factors such as genetic background, age, and ethnicity: individuals with brown eyes have much more melanin within their iris than those with blue eyes.^[^ ^68^ ^]^ This variation can result in differing rates of drug accumulation and release among patients, leading to unpredictable therapeutic outcomes. Thus, a precision medicine approach based on melanin levels may be required to achieve optimal drug dosing.

Numerous antibody‐drug conjugates (ADCs) are efficacious in the treatment of cancer; however, few ADCs have been advanced for ocular diseases and progressed to clinical trials.^[^ ^69^ ^]^ ADCs have been developed to actively target and inhibit the proliferation of epithelial and endothelial cells within the eye. This approach has been evaluated both in preclinical models and in clinical trials and most commonly for choroidal neovascularization (CNV) due to its prevalence and a broad range of target antigens. Wet AMD is distinguished by the emergence of anomalous new blood vessels originating from the choriocapillaris and extending into the retina. This causes fluid leakage, accumulation, and even hemorrhages from these abnormal vessels, which can lead to impaired vision. If left untreated, these manifestations will trigger RPE detachment, subretinal fibrosis, and RPE cell death followed by photoreceptor death, deterioration of vision, and ultimately blindness.^[^ ^70^ ^]^ Vascular endothelial growth factors (VEGF) have been unequivocally established as the primary therapeutic target for CNV. Mayo et al. utilized a photosensitizer called verteporfin conjugated with antibodies against VEGF, to increase targeting, thereby enhancing the therapeutic effect of photodynamic therapy to eliminate abnormal blood vessels in the eye.^[^ ^71^ ^]^ The conjugated verteprofin was assessed on VEGF‐expressing murine endothelial cells, both with and without subsequent laser exposure. The results showed that the conjugated verteprofin exhibited increased cellular target destruction compared to the injection of verteporfin alone. Now, verteporfin, known as Visudyne is the only ADC‐based FDA‐approved photodynamic therapy for ophthalmic use.^[^ ^72^ ^]^

Intravitreal injection of biologics, such as anti‐VEGF, has completely changed the treatment of individuals with wet AMD. There are four FDA‐approved antibodies against VEGF: pegaptanib (OSI Pharmaceuticals, Long Island, NY, USA), ranibizumab (Genentech Inc., San Francisco, CA, USA), aflibercept (Regeneron, Tarrytown, NJ, USA), and brolucizumab (Novartis, Basel, Switzerland).^[^ ^73^ ^]^ The frequent (typically monthly) treatment schedule revealed a significant unmet need in wet AMD management due to its surgical invasiveness.^[^ ^74^ ^]^ In January 2022, the FDA approved faricimab (brand name Vabysmo) a ground‐breaking bispecific monoclonal antibody. Faricimab is an insulin growth factor 1 (IGF1) bispecific antibody that targets both VEGF and angiopoietin‐2 (Ang‐2). Both pathways play roles in neovascularization and vascular leakage, with Ang‐2 contributing to increased vascular instability and exudation. With simultaneous inhibition of both pathways, more comprehensive suppression of neovascularization and exudation should result in greater vascular stability for a prolonged period of time.^[^ ^75^ ^]^ In fact, patients with wet AMD typically receive an initial series of four intravitreal injections of faricimab administered every four weeks. After this initial phase, these follow‐up injections can occur either every 8 or 12 weeks as determined by the patient's specific condition and response to treatment.^[^ ^74^ ^]^

Protein therapeutic longevity is related to target binding affinity. Aflibercept (brand name Eyelea) binds strongly to VEGF receptors (*K*_D_ = 0.5 pm) and thus lasts longer than bevacizumab, which has a 3‐orders of magnitude lower binding affinity (*K*_D_ = 58 pm).^[^ ^76^ ^]^ Treatment guidelines recommend less frequent dosing intervals of 8 to 12 weeks for aflibercept versus 4 to 6 weeks dosing for bevacizumab, reflecting the correlation between binding affinity and therapeutic longevity.^[^ ^77^ ^]^ This difference underscores the importance of molecular interactions in determining the duration and effectiveness of protein therapeutics (Figure 4C).

Designed ankyrin repeat proteins (DARPins) represent a novel class of ocular protein therapeutics that may be more effective than current ADCs. DARPins are compact and highly stable proteins, comprising engineered ankyrin repeat domains, that are synthesized via *Escherichia coli* (*E. coli*).^[^ ^78^, ^79^ ^]^ Utilizing extensive ribosome display libraries and error‐prone polymerase chain reaction, molecules with high‐affinity and high specificity binding to target proteins were designed. The binding mechanism of DARPins differs from that of conventional antibodies, contributing to their robust high affinity. Unlike antibodies that involve flexible hypervariable loops adapting to antigens, DARPin molecules boast a rigid binding surface that minimizes free entropy loss upon binding.^[^ ^80^ ^]^ Typically, DARPins exhibit picomolar binding affinities and are distinguished by their remarkable stability, in addition to exceptional affinity and selectivity. In contrast to antibodies, which have an average melting temperature *T*_m_ of ≈60‐80 °C, DARpin's have a *T*_m_ that often exceeds 80 °C and sometimes even reaches 100 °C. With a molecular mass of ≈14 to 18 kDa for a standard four‐ or five‐repeat DARPin, these molecules are around one‐tenth the size of antibodies or one‐third the size of Fab fragments.^[^ ^81^ ^]^ This smaller size facilitates higher dosing on a molar basis and the potential for improved tissue penetration. Additionally, DARPin molecules can be engineered to modulate local or systemic pharmacokinetics by incorporating extra domains or through PEGylation.^[^ ^82^ ^]^ Thus, they offer several advantages over existing antibodies or antibody fragments, including high affinity, stability, and compact size.

Potent DARPin molecules capable of neutralizing VEGF have been discovered. For example, Rodrigues et al. investigated abicipar, a 14 kDa recombinant protein linked to PEG, which is currently undergoing phase III trials for neovascular AMD treatment.^[^ ^83^ ^]^ The phase I/II dose‐escalation studies demonstrated the efficacy of abicipar for diabetic macular edema (DME) and wet AMD.^[^ ^84^, ^85^ ^]^ The pharmacokinetic data from the DME study showed that abicipar remains in the aqueous humor at levels significantly above the half maximal inhibitory concentration (IC_50_) for at least 12 weeks, requiring less frequent injections than anti‐VEGF antibodies. Abicipar exhibits high‐affinity binding to VEGF and effectively neutralizes it. Abicipar possesses similar potency to aflibercept while surpassing that of bevacizumab and ranibizumab.^[^ ^83^ ^]^ These findings collectively highlight the potential of abicipar to deliver prolonged clinical efficacy in patients with retinal neovascular disorders. However, DARPins possess some limitations as binders, including a concave binding surface, rigidity, and the limited randomization of amino acid residues in variable regions. These factors may constrain the diversity of potential target interactions.^[^ ^86^ ^]^

Affibody molecules (≈7–8 kDa) have also been investigated to overcome limitations of antibody‐based (≈150 kDa) therapies, such as limited tissue permeability. Affibodies encompass primarily one domain from a three‐helix bundle, comprising a 58‐amino acid sequence derived from a domain within staphylococcal protein A.^[^ ^87^ ^]^ Within this structure, two helices create the binding site for interactions with target molecules. By introducing randomization in 13 amino acids situated within these helices, a library was generated and serves as the foundation for generating affibody molecules that are specific with high affinity toward the desired targets, enabling the creation of novel affibody variants.^[^ ^88^ ^]^ This methodology gives rise to recombinant combinatorial affibody libraries, with an impressive diversity of sequences, reaching ≈1 × 10^11^ variations.^[^ ^89^ ^]^ Affibodies have been found for more than 40 distinct disease targets, including human epidermal growth factor receptors 2 and 3 (HER2 and HER3), tumor necrosis factor‐alpha (TNF‐α), fibroblast growth factor 2 (FGF2), pigment epithelium derived‐factor (PEDF) and IGF1.^[^ ^89^, ^90^, ^91^ ^]^ Their compact nature facilitates superior penetration within tissues compared to larger proteins, which may be advantageous for ocular administration. Additionally, their production cost is lower as affibodies are expressed in bacteria whereas antibodies are expressed in mammalian cells.^[^ ^92^ ^]^ Luong et al. engineered a novel affibody to modulate angiogenesis via the Ang2/Tie2 signaling pathway and demonstrated similar therapeutic benefit as bevacizumab alone.^[^ ^93^ ^]^ Affibody‐based therapies and drug conjugates hold promise for next‐generation, active‐targeting, therapeutic strategies for ocular treatments.

### 2.3 Drug Conjugates as their Own Delivery Vehicles

Typically, polymers like polycaprolactone (PCL) and poly(lactic‐co‐glycolic acid) (PLGA) have been used in carrier systems for controlled ocular drug delivery applications. For example, corticosteroids, which are widely used to treat ocular inflammatory conditions (e.g., keratitis, uveitis, etc.) are delivered in polymeric delivery systems to mitigate systemic side effects, such as cortisol suppression and cataracts; however, these delivery vehicles have limitations, including low drug loading (typically composed of more than 80–90% polymer by mass), initial burst, and less‐than‐optimal release kinetics where drug concentration does not maintain therapeutic levels.^[^ ^94^ ^]^ These issues can lead to excessive drug concentrations, causing local and systemic side effects and inadequate duration of dosing. One contributing factor to the unpredictable release kinetics is the bulk erosion mechanism in polymer degradation.^[^ ^95^ ^]^ In the case of PLGA‐based injectable implants, water permeates the structure, causing hydrolysis of the polymer matrix and creating pores for drug diffusion.^[^ ^96^ ^]^ The acidic degradation products of PLGA, such as lactic and glycolic acids, have been linked to chronic inflammation in ocular tissues. Additionally, remnants of drug‐free polymer can persist after the delivery is complete, contributing to toxicity concerns.^[^ ^97^ ^]^ In contrast, an ideal drug delivery system, as envisioned by Langer and Peppas in 1981, would release the drug through a surface‐mediated process only.^[^ ^98^ ^]^ This system would minimize the longevity of degradation products, thereby reducing adverse tissue reactions.^[^ ^99^ ^]^ Researchers have dedicated considerable effort to enhance drug loading in polymeric systems. For example, Snejdrova et al. used branched PLGA to encapsulate and release rifampicin to treat a non‐ocular disease and achieved loading efficiencies between 6% and 40% depending on the pH of the aqueous phase.^[^ ^100^ ^]^ While progress has been made, it is worth noting that drug loading efficiency still tends to fall below 50%. This demonstrates the persistent challenge in achieving high drug loading in nanoparticle systems and underscores the need for ongoing research and innovation, leading to more effective delivery.

Alternatively, multiple carrier‐free strategies have been investigated where the therapeutics are formulated as their own delivery vehicles. One notable example is a polymer‐free, injectable intraocular solid implant for the sustained delivery of dexamethasone (Dex) by Battiston et al.^[^ ^101^ ^]^ Specifically, they conjugated two Dex molecules with a triethyleneglycol (TEG) linker to obtain Dex dimers (Dex‐TEG‐Dex) and then formed a stand‐alone implant by extrusion through a 30‐gauge needle. In a rabbit model, they demonstrated predictable pharmacokinetics, prolonged duration of drug release, and effectiveness for up to 6 months versus Ozurdex, a prominent commercially available polymeric implant that releases dexamethasone for 2 months.^[^ ^101^ ^]^ Alternatively, Hsueh et al. formulated drug‐rich, self‐assembled ion‐complex microcrystals of sunitinib‐pamoate, which are insoluble in water. A single subconjunctival injection of these microcrystals resulted in sustained therapeutic concentrations in the retina for 20 weeks and protection of retinal ganglion cells (RGCs) in a rat model of optic nerve injury, thereby providing a promising strategy to manage glaucoma.^[^ ^102^ ^]^ Similarly, Cai et al. chemically modified dexamethasone by incorporating an acid‐sensitive component to formulate stearoxyl‐ketal‐dexamethasone pro‐drug microcrystals (SKD MCs). These SKD MCs consistently demonstrated safety and efficacy in managing uveitis inflammation in a rat model. SKD MCs exhibited significant reductions in experimental autoimmune uveitis‐related inflammation, the ability to suppress inflammatory cytokines, and protection of retinal function for at least 18 days.^[^ ^103^ ^]^

Colloidal drug aggregates (CDA) possess compelling attributes that render them promising candidates for long‐acting drug delivery formulations. CDAs are drug‐rich nanoparticles that form when hydrophobic drugs, dissolved in organic solvent, are diluted into aqueous solution above their critical aggregation concentration and self‐assemble by liquid‐liquid phase separation.^[^ ^104^ ^]^ With relatively small amounts of excipients, including polymers, proteins, lipids, or azo dyes, CDAs are stabilized. Ganesh et al. and Donders et al. demonstrated the use of proteins (i.e., bovine serum albumin, human immunoglobulin G, etc.) and lipid excipients (i.e., DSPC, cholesterol, etc.) to stabilize CDAs.^[^ ^105^, ^106^, ^107^ ^]^ While most studies have explored CDAs with chemotherapeutics, they may be equally compelling for use with ocular therapeutics.

## 3 Modifying Carrier Physicochemical Properties for Enhanced Ocular Drug Delivery

Numerous therapeutics, including hydrophilic and hydrophobic small molecules, protein biologics, and gene therapeutics require a polymeric or lipid carrier for efficacy and long‐acting effects. Thus, leveraging or modifying the physicochemical properties of injectable drug delivery vehicles presents a promising avenue for enhanced therapeutic efficacy.

### 3.1 Tuning Polymer Physicochemical Properties Prolongs Drug Pharmacokinetics

**Figure 5:**
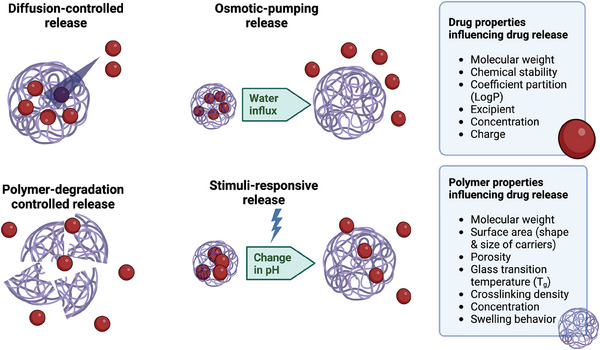
Main mechanisms and parameters of drug release from polymer‐based drug delivery vehicles. The red particles symbolize the drug, while the interconnected yarns represent the polymeric carriers.

Typically, LAIs designed for ocular drug delivery, such as biodegradable microspheres or implants, gradually release therapeutic agents over an extended period.^[^ ^108^ ^]^ These formulations exploit the unique anatomy of the eye, enabling sustained drug release directly to the intended ocular tissues. This enhances drug bioavailability, reduces the frequency of administration, and enhances patient compliance. The release kinetics depend on drug solubility, partition coefficient (LogP), molecular weight, polymer glass transition temperature (*T*_g_), molar mass, rate of degradation, and shape of the drug carrier itself (**Figure** **5**).^[^ ^109^ ^]^

Synthetic polymers, including PLGA, PLA, and PCL have been tested as LAIs for chronic ocular diseases for sustained release of small molecules, such as etoposide, lupeol, and timolol maleate.^[^ ^110^, ^111^, ^112^, ^113^ ^]^ The release from biodegradable polymeric implants typically spans from weeks to a few months and can shift as the polymer degrades. For example, Durysta is a long‐acting PLGA‐based intracameral implant for the sustained delivery of a prostaglandin analog, bimatoprost, and was the first‐in‐class, FDA‐approved in 2020 for the treatment of chronic glaucoma.^[^ ^10^ ^]^ Following intracameral administration, bimatoprost is slowly released, leading to a reduction in IOP. The implant's design ensures a consistent and controlled release over 3–4 months.^[^ ^114^ ^]^ PLGA or PLA have also been formulated as nano‐ or micro‐suspensions with drugs, such as triamcinolone acetonide and dexamethasone, and biocompatible solvent and then injected into confined ocular spaces, demonstrating sustained release for specific durations in vivo.^[^ ^115^, ^116^ ^]^ Meng et al. formulated long‐acting PLA‐based microparticles for the sustained release of dexamethasone disodium phosphate (DSP) to prevent corneal graft failure.^[^ ^117^ ^]^ They increased the encapsulation efficiency of DSP via the formation of an ionic bridge between the terminal carboxyl groups of their customized PLA and the phosphate groups from DSP using zinc. A single subconjunctival injection of PLA DSP nanoparticles effectively reversed early signs of corneal graft rejection and maintained the graft survival for at least 6 months in rats.^[^ ^117^ ^]^ To further prolong the release of hydrophobic small molecules, PCL can be leveraged to form LAIs as it is more hydrophobic than PLGA and hence degrades at a slower rate.^[^ ^118^ ^]^ Indeed, rapamycin and omidenapag isopropyl (DE‐117) were released from PCL for 16 and 24 weeks, respectively.^[^ ^20^ ^]^ While particles or injectable implants hold promise for achieving sustained release of small molecules in ocular drug delivery, hydrogels remain the preferred choice for delivering protein biologics to the eye^[^ ^119^ ^]^ because proteins are often degraded by the organic solvents and high shear forces used in polymeric nano/micro‐encapsulation.^[^ ^120^ ^]^ Alternatively, some researchers have explored the use of natural‐synthetic copolymers to create LAI solutions, aiming to mitigate potential toxicity associated with certain synthetic polymers. For example, Luo et al. developed an innovative approach with chitosan‐*g*‐poly(N‐isopropylacrylamide) where 4‐hydroxy‐3,5‐dimethoxybenzoic acid was delivered to provide potent antioxidant properties for the treatment of glaucomatous nerve damage.^[^ ^121^ ^]^ Using New Zealand white rabbits, they co‐loaded the thermogel with pilocarpine and RGFP966 and injected it intracamerally, resulting in remarkable improvements in countering neurodegeneration by suppressing oxidative stress, lowering ocular hypertension, reducing retinal ganglion cell loss, and promoting myelin growth and neuron regeneration for up to 70 days.

Long‐acting release of proteins from both naturally‐derived and synthetic polymeric hydrogels has been demonstrated. Natural polymers, such as hyaluronic acid (HA), and gelatin, are enzymatically degraded and minimize tissue irritation and chronic inflammation. These polymers mimic the natural extracellular matrix, facilitating cell interaction, migration, and tissue regeneration.^[^ ^122^, ^123^ ^]^ Their hydrophilic nature promotes water retention and solubility of protein biologics, maintaining the conformational stability critical for therapeutic efficacy.^[^ ^124^, ^125^ ^]^ While conventional hydrogels rely solely on diffusion for protein release, affinity‐based hydrogels utilize a reaction‐diffusion mechanism driven by ligand‐protein interactions.^[^ ^126^ ^]^ These affinity‐based hydrogels are designed with ligands for protein recognition or immobilization, resulting in both free and bound protein states that dictate release profile. Unlike traditional hydrogels, affinity hydrogels involve protein‐ligand binding reactions, thereby slowing the rate of protein release.^[^ ^127^ ^]^ One example is heparin‐based hydrogels, which draw inspiration from the innate affinity of numerous proteins for the extracellular matrix (ECM). Heparin, a large electronegative protein, exhibits moderate to high affinity (*K*_D_ ≈10^−6^–10^−9^ m) for growth factors primarily through electrostatic interactions. Heparin has been chemically bound to natural polymers using carbodiimide chemistry.^[^ ^128^ ^]^ For example, Xu et al. developed heparin‐functionalized poloxamer based‐hydrogels to sustain the release of FGF2 up to 7 days.^[^ ^129^ ^]^ Peptide‐ or aptamer‐based affinity hydrogels have also been used to prolong the release of ocular therapeutics.^[^ ^130^, ^131^, ^132^ ^]^ For example, Delplace et al. designed an intravitreal, affinity‐based release system for ciliary neurotrophic factor (CNTF) as a model biologic.^[^ ^133^ ^]^ To prolong the release of CNTF, it was expressed as a fusion protein with Src homology 3 (SH3) and released from a hyaluronan and methylcellulose thermogel (HAMC) that was modified with SH3 binding peptides. SH3‐CNTF was released over 7 days in vitro and demonstrated prolonged bioactivity by downregulating phototransduction genes in the retina of mice after intravitreal delivery.^[^ ^133^ ^]^ Affibody‐mediated release from hyaluronan‐based hydrogels has been achieved with multiple protein biologics, including IGF1, PEDF, and FGF2.^[^ ^89^, ^91^ ^]^ Specifically, Teal et al. demonstrated the potential not only to tune the release kinetics of a protein therapeutic from an HA‐based hydrogel but to enhance its biological activity in vivo. Using affibody binding partners with moderate affinity and high specificity to each of IGF1 and PEDF, both proteins were simultaneously released at distinct rates over 7 days in vitro. In vivo studies in mice demonstrated prolonged activity of IGF1 through affibody‐controlled release versus bolus injection, thereby illustrating a versatile method for precise and independent control of ocular therapeutic proteins.^[^ ^91^ ^]^ Similarly, Bostock et al. demonstrated the ability to prolong the release of FGF2 over 7 days in vitro from a crosslinked HA‐based hydrogel. Importantly, the FGF2‐binding affibody stabilized FGF2 structure, thereby opening opportunities for the delivery of other inherently unstable biologics.^[^ ^89^ ^]^ Small‐molecule drugs present their own challenges when using hydrogels due to their reduced size compared to proteins. Thus, a hydrogel‐nanoparticle composite system is often employed to enhance the precision of drug release rates and enable sustained release. For example, Meany et al. used a supramolecular polymer‐nanoparticle (PNP) hydrogel for intravitreal delivery of bimatoprost. The injectability of PNP hydrogels can be attributed to their shear‐thinning and self‐healing properties, and in vivo studies in rabbits demonstrated extended drug release for up to 8 weeks.^[^ ^134^ ^]^ Lastly, specific requirements for hydrogels as a long‐term drug delivery carriers include factors such as injectability and degradability. Achieving hydrogel injectability or in situ gelation is crucial considering the turnover rate of fluids in the eye. To meet this requirement, Baker et al. designed a newly engineered hydrogel vitreous substitute using hydrolytically stable oxime chemistry. By combining hyaluronan‐aldehyde, hyaluronan‐ketone, and PEG‐oxyamine in a click‐crosslinked system, this hyaluronan‐oxime rapidly gelled and remained injectable. The hydrogel was shown to be biodegradable and biocompatible in New Zealand white rabbits over 56 days, and showed promise for maintaining retinal function.^[^ ^135^ ^]^ These results underscore the importance of appropriate properties for hydrogels, such as injectability, controlled swelling, and biocompatibility, in their role as potential vitreous substitutes and long‐term drug delivery carriers.

Lipid‐based formulations have garnered substantial attention as promising vehicles for sustained release. For example, Natarajan et al. developed a liposomal formulation to prolong the release of latanoprost, a prostaglandin analog, for the treatment of glaucoma. Through a single subconjunctival injection in rabbit eyes, they demonstrated a sustained reduction in IOP over 50 days comparable to that achieved by daily ocular administration.^[^ ^136^, ^137^ ^]^ This long‐acting liposomal formulation, Lipolat, is now in Phase II clinical trials. Contrary to polymeric‐based formulations, lipid‐based strategies such as liposomes are transparent, which can be important in ocular applications, depending on the site of injection.^[^ ^138^ ^]^ While liposomes prolong release, they can also suffer from premature leakage of encapsulated drugs, thereby compromising the duration of delivery. Additionally, liposomes are inherently constrained by drug loading capacity and suffer from inherent instability, including aggregation and degradation over time.^[^ ^139^ ^]^

### 3.2 Enhancing Nanoparticle Intracellular Uptake for Increased Therapeutic Effect

Gene therapy has emerged as a transformative strategy in the field of ocular therapeutics, offering unprecedented opportunities to address genetic mutations and disorders and potentially superior effects compared to conventional treatments.^[^ ^140^ ^]^ Nucleic acids (e.g., siRNA, mRNA, etc.) are negatively charged and hydrophilic, preventing their penetration through the cellular membrane. Additionally, they are susceptible to enzymatic degradation by nucleases present in the extracellular environment.^[^ ^141^, ^142^ ^]^ Nanocarriers have been designed to facilitate the precise and efficient delivery of therapeutic genes. These nanoscale delivery systems, often composed of lipids or polymeric materials, encapsulate and protect therapeutic nucleic acids, enhancing their stability and facilitating targeted delivery to specific ocular tissues. Additionally, nanocarriers can be functionalized with ligands or antibodies that specifically recognize cell surface receptors overexpressed in diseased cells.^[^ ^143^ ^]^ To date, lipid nanoparticles (LNPs) stand as the most advanced platform in clinical applications for the delivery of RNA therapeutics. The widespread use of LNPs in the context of the COVID‐19 mRNA vaccines serves as a testament to their demonstrated safety and effectiveness.^[^ ^144^ ^]^ Numerous studies have focused on LNP‐mediated RNA delivery for inherited retinal degenerations (IRDs). This category encompasses a complex array of genetic disorders that emanate from mutations within more than 300 distinct genes associated with retinal pathology. While lipid nanoparticles are an effective delivery strategy, functionalization is often needed for active targeting.^[^ ^145^ ^]^

LNPs are unable to penetrate the neural retina when delivered intravitreally or subretinally.^[^ ^146^ ^]^ Herrera‐barrera et al. investigated the use of peptide ligands that target photoreceptors to deliver mRNA therapeutics for the treatment of IRDs.^[^ ^147^ ^]^ Using a bacteriophage‐based peptide library, they identified peptide sequences that bind the neural retina in vivo and chemically synthesized the peptide conjugates of interest, before functionalizing them on the surface of LNPs. They demonstrated effective delivery of mRNA to the photoreceptors in mice and non‐human primates.^[^ ^147^ ^]^ The surface charge of nanocarriers has also been shown to play a critical role in altering the transport of RNA therapeutics through ocular membranes. By incorporating an amine‐acylate lipidoid (i.e., 306O_13_), Huang et al. showed that positively charged LNPs, with a zeta potential of ≈+30–40 mV, diffused effectively across the retina to deliver siRNA to the RGCs and the inner plexiform layer (IPL) following intravitreal injection in mice.^[^ ^148^ ^]^

Overexpressed receptors, due to ocular pathologies, can be leveraged for active targeting of nanocarriers.^[^ ^149^ ^]^ Previous research has shown that trabecular meshwork (TM) cells from patients with glaucoma overexpress CD44 cell‐surface receptors,^[^ ^150^ ^]^ which can be targeted with HA‐coated nanoparticles.^[^ ^151^ ^]^ Dilinger et al. utilized HA‐coated PLGA nanoparticles conjugated with branched polyethylenimine (bPEI) to encapsulate siRNA of connective tissue growth factor (CTGF) and demonstrated effective uptake and delivery in human glaucomatous trabecular meshwork cells that overexpress CD44.^[^ ^149^ ^]^ Despite a plethora of research exploring the potential of gene therapy in ocular delivery, only Voretigene neparvovec, commercially known as Luxturna, is clinically approved and uses adeno‐associated virus (AAV) to repair the RPE65 gene mutation in inherited retinal dystrophy.^[^ ^152^ ^]^ Notwithstanding this great achievement, AAV‐based therapies often possess risks of intrinsic immunogenicity against the capsid, which hinders their dose of administration in the medium‐ and long‐term treatment.^[^ ^153^ ^]^

## 4 Challenges and Opportunities for Ocular LAIs

Due to the outstanding progress in developing delivery formulation designs for ocular diseases, LAIs in clinical practice have grown since Ozurdex, which was the first ocular FDA‐approved LAI in 2009 for the treatment of macular edema and uveitis. One of the most significant advantages of LAIs for ocular diseases is their potential to significantly improve treatment. Patients often struggle with adhering to frequent eye drop regimens, leading to suboptimal outcomes. LAIs address this challenge by providing a controlled and prolonged release of medication at the local therapeutic target, reducing the need for frequent administration. Many additional LAI products are currently in the developmental pipeline. One of the foremost technical challenges within these formulations pertains to determining the appropriate dosage magnitude and release duration. These factors are intricately linked with the drug's potency and the constraints imposed by injection volume limitations in the ocular space. A secondary, yet substantial hurdle, involves safeguarding the physical and chemical stability of proteins and other biologics during both microencapsulation and subsequent release. Importantly, the realm of possibilities for LAIs is rapidly growing.

While the concept of *long‐acting* may intuitively seem advantageous in the context of ocular drug delivery, it is important to recognize that *longer* does not necessarily equate to *better*. Achieving an optimal balance between drug release duration and therapeutic effectiveness is a complex task that requires careful consideration of many factors (listed in **Table** **2**). Many research communities and pharmaceutical developers tend to focus on extending drug retention through prolonged drug release or active drug targeting without a comprehensive understanding of the specific design requirements dictated by each disease or condition being treated. This approach can lead to misaligned expectations and suboptimal outcomes. The pursuit of longer drug release in long‐acting ocular drug delivery must be guided by a patient‐centric and disease‐specific approach. Achieving the right balance between therapeutic efficacy and safety, patient compliance, and practicality are key determinants for success.

**Table 2:** Challenges to overcome for designing an ideal LAI.

Challenges	Comments
Drug Loading and Release	Enhancing the drug loading capacity of LAIs, especially for small molecules, while maintaining controlled, sustained release profiles is a significant challenge.
Injectability and Gelation Control	Designing LAIs that are easy to inject into the eye and can undergo controlled gelation post‐injection is crucial for patient comfort and safety.
Biocompatibility	Ensuring that LAIs do not trigger adverse immune responses, inflammation, or discomfort in the eye is vital.
Stability	Developing LAIs that remain stable and maintain drug release over extended periods without degradation or unintended swelling is essential for their long‐term effectiveness.
Patient Compliance	Addressing the need for patient‐friendly LAIs that reduce the frequency of administration, improving patient adherence and comfort.
Specific Disease Targeting	Tailoring LAIs for specific ocular diseases and their unique requirements, such as controlled release in the retina for conditions like age‐related macular degeneration.
Biodegradability	Designing LAIs that can be safely absorbed or removed from the eye when the treatment is completed.
IOP	Preventing potential increases in intraocular pressure, which can lead to glaucoma or other complications

Although the safety of short‐acting ocular therapies is well‐defined, the field of ocular toxicology underscores the unique challenges and complexities involved in assessing the safety long‐acting formulations. The prolonged presence of formulations within the eye and their potential to induce distinctive toxicities necessitates attention. Thackaberry et al. underlined the limited existing knowledge regarding the ocular toxicity of substances administered via these routes, thus necessitating the safety assessment of sustained‐release therapies. To improve nonclinical toxicology studies of LAIs, five major categories of toxicities, including local chemical‐mediated, local immune‐mediated, local physical‐mediated, ocular phototoxicity, and systemic (extraocular) toxicities.^[^ ^154^ ^]^ A robust method of evaluating the ocular safety of LAIs is key to their effective clinical translation.

Furthermore, long‐acting eye drops have recently witnessed substantial growth, primarily due to their inherent ease of use by patients. This non‐invasive approach to drug delivery offers several advantages over traditional LAIs by eliminating the need for injections, which can be uncomfortable and inconvenient for patients. Additionally, non‐invasive long‐acting eye drops reduce the risk of infection associated with injections, making them safer for long‐term treatments. Eye drops are self‐administered, giving patients greater control over their treatment regimen. These formulations can be more cost‐effective, potentially leading to greater accessibility. For example, Luo et al. developed an innovative, long‐acting topical treatment for dry eye disease (DED) by combining gelatin for enzymatic degradation, poly(N‐isopropylacrylamide) for controlled release, and Helix pomatia agglutinin for mucoadhesion. In a rabbit model of DED, a single topical application of epigallocatechin gallate‐loaded carriers effectively repaired corneal epithelium, reducing inflammation, oxidative stress, and cell apoptosis for an extended 14‐day period.^[^ ^155^ ^]^ Additionally, Lai et al. formulated a novel hydrogel eye drop with strong mucoadhesive, tight‐junction opening, and antioxidant properties, for glaucoma treatment. Using glutathione, a single topical administration of the hydrogel eye drop enhanced pilocarpine bioavailability in a rabbit model of glaucoma.^[^ ^156^ ^]^ Thus long‐acting eye drops may ultimately be useful to help manage ocular diseases; however, the challenge of self‐application and prolonged effects are non‐trivial to overcome.

Given the fast‐growing development of new LAIs entering the market, it is evident that they are and will play a significant role in patient care. The horizon for treating chronic diseases appears promising, offering the potential for personalized, remarkably efficient, and safe LAIs that revolutionize the way we manage ocular diseases.

## Conflict of Interest

The authors declare no conflict of interest.
